# Severe 2010 Cold-Water Event Caused Unprecedented Mortality to Corals of the Florida Reef Tract and Reversed Previous Survivorship Patterns

**DOI:** 10.1371/journal.pone.0023047

**Published:** 2011-08-10

**Authors:** Diego Lirman, Stephanie Schopmeyer, Derek Manzello, Lewis J. Gramer, William F. Precht, Frank Muller-Karger, Kenneth Banks, Brian Barnes, Erich Bartels, Amanda Bourque, James Byrne, Scott Donahue, Janice Duquesnel, Louis Fisher, David Gilliam, James Hendee, Meaghan Johnson, Kerry Maxwell, Erin McDevitt, Jamie Monty, Digna Rueda, Rob Ruzicka, Sara Thanner

**Affiliations:** 1 Rosenstiel School of Marine and Atmospheric Science, University of Miami, Miami, Florida, United States of America; 2 Atlantic Oceanographic and Meteorological Laboratory, National Oceanic and Atmospheric Administration, Miami, Florida, United States of America; 3 National Oceanic and Atmospheric Administration, Florida Keys National Marine Sanctuary, Key Largo, Florida, United States of America; 4 Institute for Marine Remote Sensing/IMaRS, College of Marine Science, University of South Florida, St. Petersburg, Florida, United States of America; 5 Broward County Natural Resources Planning and Management Division, Plantation, Florida, United States of America; 6 Center for Coral Reef Research, Mote Marine Laboratory, Summerland Key, Florida, United States of America; 7 Biscayne National Park, Homestead, Florida, United States of America; 8 The Nature Conservancy, Summerland Key, Florida, United States of America; 9 National Oceanic and Atmospheric Administration, Florida Keys National Marine Sanctuary, Key West, Florida, United States of America; 10 Florida Park Service, Islamorada, Florida, United States of America; 11 Broward County Environmental Protection and Growth Management Department, Plantation, Florida, United States of America; 12 National Coral Reef Institute, Nova Southeastern University Oceanographic Center, Dania Beach, Florida, United States of America; 13 Florida Fish and Wildlife Conservation Commission, Fish and Wildlife Research Institute, Marathon, Florida, United States of America; 14 Florida Fish and Wildlife Conservation Commission, West Palm Beach, Florida, United States of America; 15 Florida Department of Environmental Protection, Coral Reef Conservation Program, Florida Gulf Coast University, Miami, Florida, United States of America; 16 Fish and Wildlife Research Institute, St. Petersburg, Florida, United States of America; 17 Department of Environmental Resources Management, Miami, Florida, United States of America; Institute of Marine Research, Norway

## Abstract

**Background:**

Coral reefs are facing increasing pressure from natural and anthropogenic stressors that have already caused significant worldwide declines. In January 2010, coral reefs of Florida, United States, were impacted by an extreme cold-water anomaly that exposed corals to temperatures well below their reported thresholds (16°C), causing rapid coral mortality unprecedented in spatial extent and severity.

**Methodology/Principal Findings:**

Reef surveys were conducted from Martin County to the Lower Florida Keys within weeks of the anomaly. The impacts recorded were catastrophic and exceeded those of any previous disturbances in the region. Coral mortality patterns were directly correlated to in-situ and satellite-derived cold-temperature metrics. These impacts rival, in spatial extent and intensity, the impacts of the well-publicized warm-water bleaching events around the globe. The mean percent coral mortality recorded for all species and subregions was 11.5% in the 2010 winter, compared to 0.5% recorded in the previous five summers, including years like 2005 where warm-water bleaching was prevalent. Highest mean mortality (15%–39%) was documented for inshore habitats where temperatures were <11°C for prolonged periods. Increases in mortality from previous years were significant for 21 of 25 coral species, and were 1–2 orders of magnitude higher for most species.

**Conclusions/Significance:**

The cold-water anomaly of January 2010 caused the worst coral mortality on record for the Florida Reef Tract, highlighting the potential catastrophic impacts that unusual but extreme climatic events can have on the persistence of coral reefs. Moreover, habitats and species most severely affected were those found in high-coral cover, inshore, shallow reef habitats previously considered the “oases” of the region, having escaped declining patterns observed for more offshore habitats. Thus, the 2010 cold-water anomaly not only caused widespread coral mortality but also reversed prior resistance and resilience patterns that will take decades to recover.

## Introduction

Coastal ecosystems have undergone recent world-wide declines in response to both natural and human stressors [1]–[5]. These declines threaten both the ecological integrity of these systems as well as the livelihoods of millions of people that depend on the services provided by mangrove, seagrass, and coral reef ecosystems [6]–[8]. Coral reefs have been particularly affected by the combination of large-scale natural events such as warm-water anomalies, disease outbreaks, and major storms, as well as the chronic, localized impacts of coastal development, pollution, and over-exploitation, which have been linked to widespread coral mortality. Warm-water anomalies that can cause severe coral bleaching (i.e., the expulsion of dinoflagellate symbionts from the coral host) are one of the most important factors responsible for the demise of coral reefs. Warm-water events may influence the long-term persistence of these diverse and productive environments, especially under future global climate change-scenarios of increased temperatures [9]. However, for corals living on marginal or extreme environments, exposure to unusually cold or highly variable temperatures may be just as detrimental [10]–[12].

In January 2010, the reefs of the Florida Reef Tract (FRT) were impacted by an unusual cold-water event that caused unprecedented, large-scale patterns of coral mortality and immediate and dramatic losses in coral cover directly related to the minimum temperatures experienced as well as exposure times. This unusual climatic event was associated with extremely negative values of the North Atlantic Oscillation (NAO) index that produced northerly surface wind anomalies and the southward advection of the cold Arctic air [13]. In fact, daily temperatures lower than the minimum temperature (1.67°C) recorded at the Miami airport on January 10, 2010, were only observed during 11 days in the past 60 years in the region. During the 2010 anomaly, we observed species-specific susceptibilities that were distinct and even opposite from those recorded during warm-water anomalies, ultimately resulting in the mortality of coral species and populations that had survived recent bleaching episodes. This is an especially catastrophic outcome for a system that has already undergone a highly publicized decline over the past 20–30 years from multiple and often interacting disturbances that include coral disease outbreaks, five warm-water coral bleaching events, the demise of the sea-urchin *Diadema antillarum*, and multiple major storms and hurricanes [12], [14]–[19].

While rare, cold-water “coral kills” have been documented in Florida in the past. Shinn [20] reported mortality of *Acropora cervicornis* in shallow reefs as temperatures dropped to 13.3°C in 1962. Hudson et al. [21] and Hudson [22] documented mortality of *Montastraea* colonies in nearshore patch reefs due to cold fronts in 1969–1970 and 1977–1978 respectively. Porter et al. [23] and Davis [24] reported high coral mortality in the Dry Tortugas following severe cold temperatures (<14°C) during the 1976–1977 winter. Finally, Walker et al. [25] observed coral kills in response to cold temperatures in Florida in 1981. The 2010 cold-water anomaly represents the first time that cold-water mortality of corals was documented in Florida at a regional scale.

**Figure 1:**
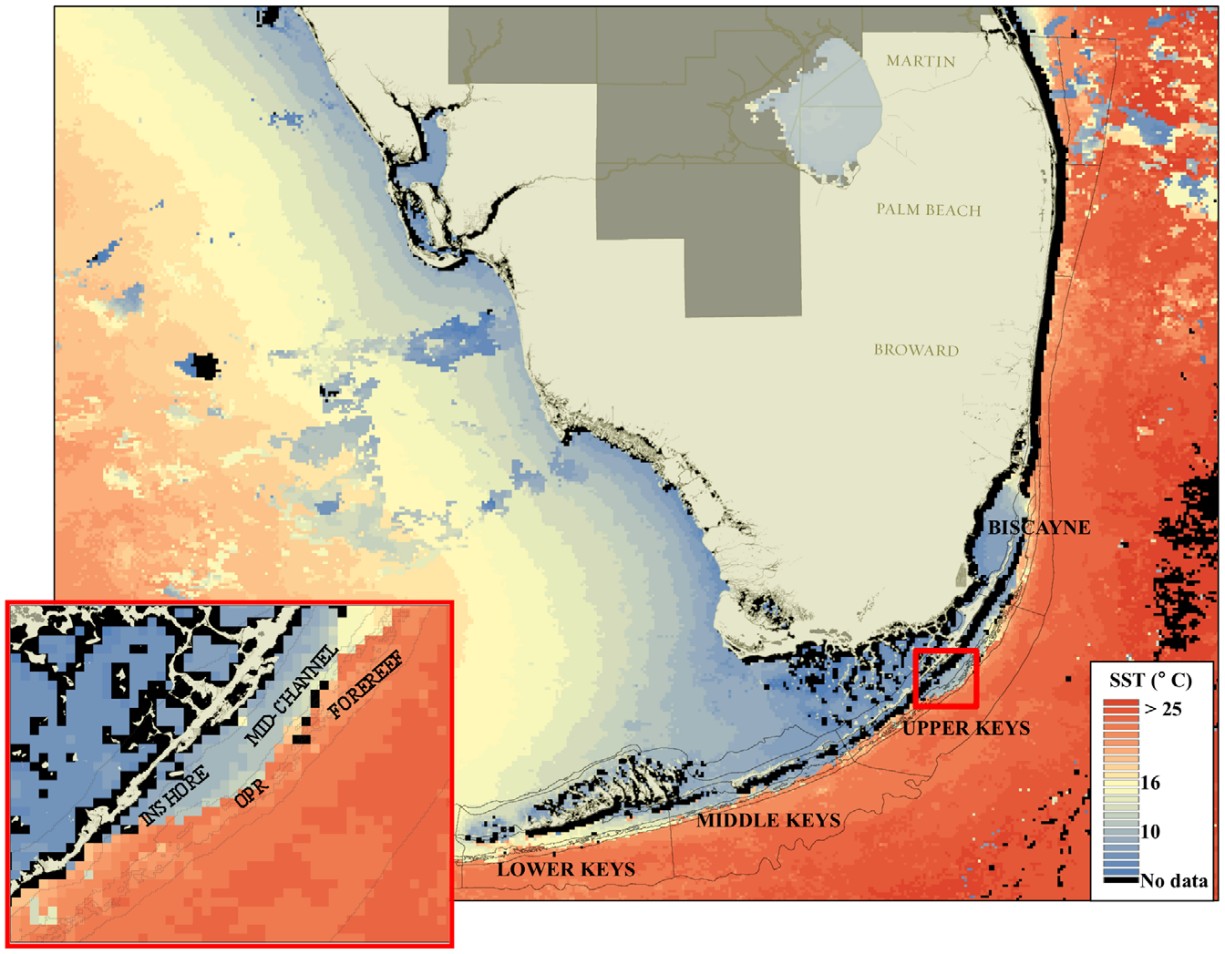
Minimum sea surface temperatures (SST) recorded during the cold-water anomaly of January 2010 in the Florida Reef Tract. The insert shows the different habitats sampled (OPR = Offshore Patch Reefs). SST values were obtained using the AVHRR satellite sensor (F. Muller-Karger, USF).

Temperature has long been considered one of the primary factors controlling reef distribution [26]–[28], with the optimum temperature for coral growth around 26–27°C [29]–[31]. The present-day global distribution of coral reefs generally coincides with the 18°C monthly minimum seawater isotherm [32], [33]. Cold water can influence coral photosynthetic efficiency of endosymbiotic dinoflagellates and metabolism of the coral holobiont, result in bleaching, and, ultimately, coral mortality [11], [34]. Cold-temperature tolerances are not well defined for corals, but early experiments showed that prolonged exposure to 16°C is stressful to most species and that exposure to temperatures <14°C for as little as 9 hrs can result in coral mortality [35]–[37]. During the January 2010 cold-water event, water temperatures <16°C were recorded for up to 6 days on the Florida Reef Tract (Figure 1), causing significant, rapid mortality of most coral taxa, underscoring the role that rare, but severe, acute disturbances can have on the survivorship of corals and coral reefs. In this study, we describe the activities and outcome of a rapid-response program, the Florida Reef Resilience Program (FRRP) that, through the coordinated effort of multiple stakeholders characterized the January 2010 cold-water anomaly as one of the worst disturbances to impact the FRT in recorded history.

## Results

The comparison of recent coral tissue mortality patterns between January 2010 and all other surveys since 2005 highlights the impacts of the extreme-cold event and reveals differences in coral response based on type of thermal stress (warm vs. cold), species, and temperature patterns. Mean percent tissue mortality for all corals combined was two orders of magnitude higher shortly after the cold-water event than in all previous surveys (Table 1). The mean percent coral mortality recorded for all species, subregions, and all summers combined, including years like 2005 where bleaching was prevalent, was 0.5% (S.D. = 4.4) compared to 11.5% (28.9) mortality recorded in the 2010 winter. Mean percent recent tissue mortality values for all colonies combined were significantly higher after the January 2010 cold event compared to all other years, which were not significantly different from each other (ANOVA, p<0.001). The percentage of colonies (all species and all areas combined) that exhibited complete recent mortality (100% tissue dead in response to the cold) never exceeded 0.7% in 2005–2009, whereas 7.8% of all colonies sampled in 2010 were completely devoid of live tissue.

**Table 1:** Mean percent recent tissue mortality (± S.D) of stony corals from the Florida Reef Tract.

Subregions	2005	2006	2007	2008	2009	2010
Martin	0.1 (0.6)	4.6 (16.9)	nd	0.6 (1.8)	0	**0**
Palm Beach	0.8 (2.9)	1.4 (11.9)	1.0 (5.0)	1.9 (9.7)	0.3 (1.7)	**0.1 (0.8)**
Broward	0.8 (4.0)	0.9 (6.1)	2.0 (7.6)	0.8 (4.6)	1.1 (6.5)	**0.4 (2.6)**
Biscayne	0.4 (6.1)	0.7 (5.5)	0.7 (4.7)	0.5 (4.7)	0.8 (6.0)	**5.9 (18.2)**
Upper Keys	0.3 (3.2)	0.4 (3.7)	0.3 (3.4)	0.4 (3.4)	0.2 (2.0)	**26.0 (40.4)**
Middle Keys	0.7 (6.6)	0.4 (3.5)	0.5 (3.9)	0.4 (4.4)	0.2 (1.7)	**17.6 (34.5)**
Lower Keys	0.4 (3.7)	0.5 (4.5)	0.3 (2.9)	0.3 (3.2)	0.2 (2.4)	**6.8 (22.9)**
All Subregions	0.5 (4.4)	0.7 (5.5)	0.6 (4.3)	0.5 (4.2)	0.4 (3.9)	**11.5 (28.9)**

Data combined for all corals and reef zones. Data from 2005–2009 were collected during the summer temperature peaks (Aug–Oct). Data from 2010 were collected in January–February. nd = no data available. The number of sites (and number of coral colonies) surveyed were: 2005 (96 sites, 3512 colonies), 2006 (123, 4765), 2007 (131, 6041), 2008 (209, 10408), 2009 (223, 11379), and 2010 (76, 3636).

Recent tissue mortality patterns during the 2010 cold-water event were not spatially uniform; mean mortality was highest in the Upper and Middle Keys and was less severe with increasing distance from these sites. No mortality was recorded in Martin County, the northernmost subregion surveyed (Table 1). Mean tissue mortality levels in 2010 were significantly higher than in any of the previous periods for Biscayne and all three subregions of the Florida Keys (all habitats combined, ANOVA, p<0.001). Maximum prevalence of complete colony mortality was recorded for the Middle Keys and the Upper Keys, with 17.1% and 12.6% of colonies with complete recent mortality respectively. The three northern subregions (Martin, Palm Beach, and Broward) did not exhibit significant tissue mortality related to the cold-water anomaly (ANOVA, p>0.05) and were thus removed from subsequent analyses. In all four affected regions, mortality levels were significantly higher on inshore reef habitats and diminished with increasing distance from shore and increasing depth (Figure S1). The highest levels of recent tissue mortality were documented for inshore habitats in the Middle Keys (39.1%, 47.8), followed by the Upper Keys (34.0%, 40.1), Lower Keys (23.2%, 38.2), and Biscayne (15.0%, 33.0).

Recent percent tissue mortality levels were higher in the 2010 winter surveys than all previous summers (2005–2009) for 24 of the 25 most abundant coral species (Table 2). Increases in mortality during the 2010 sampling were significant for 21 of the 25 species (t-tests, p<0.01), and were 1–2 orders of magnitude higher for most species. The most affected coral taxa were *Porites astreoides*, *P. furcata*, all species of the genus *Montastraea*, *Diploria clivosa*, and *D. strigosa*. Surprisingly, the response of corals to warm-water anomalies (data from the peak summer temperature periods in 2005–2009) was a poor predictor of the response to cold-water (linear regression of species susceptibility ranks, p>0.1). In fact, several of the species that have previously exhibited the most resistance to high temperature were most affected by the cold (Table 2).

**Table 2:** Abundance and partial mortality patterns for stony corals.

		Mean % Mortality (S.D.)			Mortality Ranking		
Species	N colonies	Summers	Winter 2010	p value	Summers	Winter 2010	Ranking Change
*Siderastrea siderea*	10329	0.2 (2.4)	1.0 (7.2)	*	20	22	N
*Porites astreoides*	5962	0.5 (4.7)	29.2 (43.8)	*	11	**4**	I
*Stephanocoenia intersepta*	4286	0.3 (3.4)	0.8 (5.6)	*	14	24	D
*Agaricia agaricites*	2588	0.4 (3.6)	7.4 (20.3)	*	13	15	N
*Montastraea cavernosa*	2210	0.3 (3.1)	31.7 (38.8)	*	18	**3**	I
*Porites porites*	2176	1.0 (6.0)	16.2 (33.1)	*	**3**	9	D
*Dichocoenia stokesi*	1571	0.2 (2.2)	1.7 (10.6)	*	19	19	N
*Siderastrea radians*	943	0.6 (6.5)	3.2 (15.9)	*	7	17	D
*Montastraea faveolata*	840	0.3 (2.8)	37.0 (41.6)	*	16	**2**	I
*Colpophyllia natans*	642	0.3 (3.5)	12.2 (29.8)	*	15	12	N
*Porites furcata*	526	0.9 (6.1)	20.7 (36.8)	*	**5**	7	N
*Solenastrea bournoni*	391	1.0 (7.8)	1.5 (5.3)	ns	**4**	20	D
*Diploria strigosa*	372	0.2 (1.9)	21.0 (32.0)	*	22	6	I
*Montastraea annularis*	343	0.3 (1.8)	56.4 (42.9)	*	17	**1**	I
*Porites divaricata*	280	1.5 (9.2)	1.95 (5.1)	ns	**2**	18	D
*Diploria labyrinthiformis*	271	0.1 (0.9)	16.7 (33.3)	*	25	8	I
*Montastraea franksi*	254	0.4 (3.6)	7.9 (17.8)	*	12	14	N
*Eusmilia fastigiata*	228	0.5 (4.8)	1.2 (3.3)	ns	10	21	D
*Meandrina meandrites*	211	0.2 (1.7)	11.1 (28.4)	*	21	13	I
*Oculina sp.*	204	0.1 (0.9)	0.8 (2.0)	*	24	23	N
*Diploria clivosa*	199	0.7 (3.9)	26.6 (40.7)	*	6	**5**	N
*Mycetophyllia sp.*	195	0.6 (6.4)	15.0 (28.3)	*	8	11	N
*Agaricia lamarcki*	116	0.1 (1.1)	4.8 (11.2)	*	23	16	I
*Acropora cervicornis*	101	2.2 (10.2)	15.4 (37.5)	*	**1**	10	D
*Madracis decactis*	96	0.6 (3.5)	0.2 (0.4)	ns	9	25	D

Only the 25 most abundant species (all sample dates from 2005–2010 combined) are included. Mortality values were compared between summers (all years combined) and winter 2010 surveys (t-test).

* = p<0.01, ns = not significant (p>0.05). Species rankings were calculated for each time interval (summers, winter 2010) from 1 (highest mortality) to 25 (lowest mortality). The top 5 most susceptible species based on recent mortality are highlighted in bold for each survey period. Changes in ranking represent changes in species susceptibility between warm-water (summers) and cold-water (winter 2010) anomalies. Ranking-change colors are changes in mortality rankings for each species between periods. D = decrease in ranking of >3 ranking spots, I = increase in ranking of >3 ranking spots, N = change in ranking of <3 spots.

The impacts of the cold event were not uniform across colony sizes and smaller colonies experienced higher levels of tissue mortality compared to larger colonies. The average size of colonies that experienced partial or complete tissue mortality was significantly larger than that of colonies that did not experience any tissue mortality for 11 of the 15 most abundant species (t-test, p<0.1) (Table S1).

**Figure 2:**
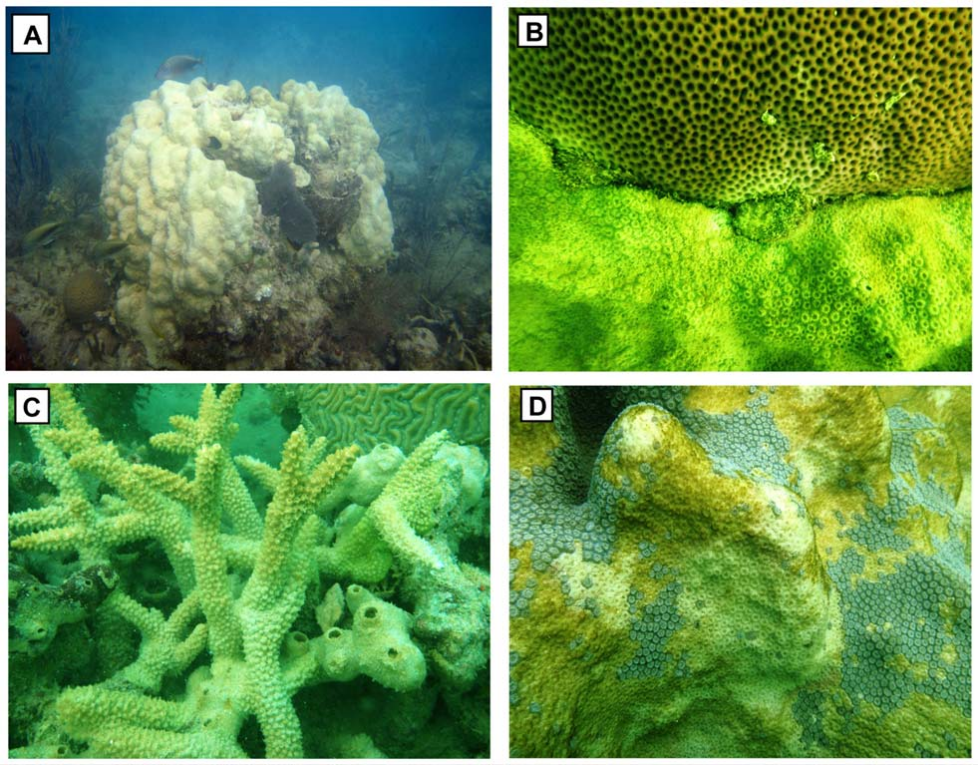
Coral colonies impacted by the January 2010 cold-water event. (A) large colony of *Montastraea faveolata* showing 100% tissue mortality due to the cold, (B) *Siderastrea siderea* colony showing no signs of mortality adjacent to a completely dead *Montastraea* colony, (C) colonies of the threatened species *Acropora cervicornis* showing 100% mortality at an inshore patch reef,(D) close-up of a colony of *M. faveolata* showing patches of surviving tissue surrounded by dead tissue. Photo credits: (A) W. Precht, (B–D) K. Maxwell.

The 2010 extreme-cold event was particularly detrimental to the genus *Montastraea*, one of the key reef-building taxa in the region (Figure 2). Mean tissue mortality of *M. annularis* was 56.4%, followed by *M. faveolata* (37.0%), *M. cavernosa* (31.7%), and *M. franksi* (7.9%). Mean tissue mortality for these taxa during the 2005–2009 summers never exceeded 0.4% (Table 2). In contrast, coral species that are dominant in both reef and shallow hard-bottom habitats of coastal bays where they are commonly exposed to wide temperature fluctuations experienced limited mortality during the 2010 event (<2%). These cold-resistant species include *Siderastrea radians*, *Stephanocoenia intersepta*, *Solenastrea bournoni*, and *Porites divaricata.* Genera like *Oculina* that are also found in deeper, colder habitats did not suffer significant losses in 2010. Species with high resistance to both warm and cold-water anomalies included *Siderastrea siderea*, *Dichocoenia stokesi*, and *Agaricia lamarcki* (Table 2).

**Figure 3:**
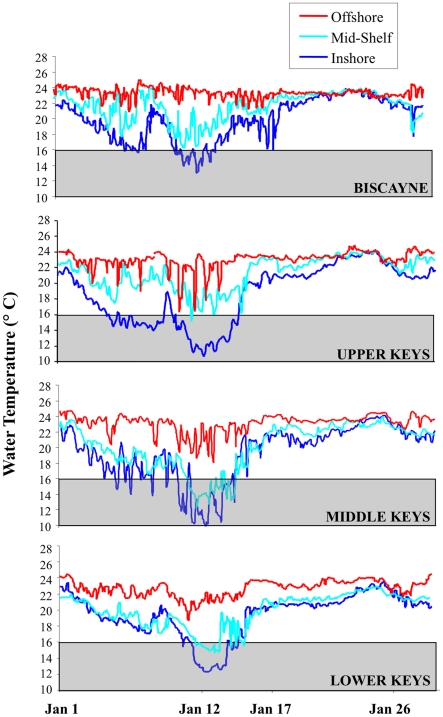
Hourly sea temperatures from representative reef sites during January 2010. The grey boxes represent the reported cold-water temperature threshold (<16°C) for corals.

*In situ* temperature patterns showed distinct differences based on region and habitat/cross-shelf location (Table 3, Figure 3). Water temperatures in reef habitats in Broward and Palm Beach Counties did not fall below 16°C during the cold anomaly, while temperatures <16°C and <14°C were recorded for 54 and 26 hrs respectively on reefs of Martin County (Table 3). The minimum temperature recorded during January 2010 was 9.5°C at inshore habitats of the Middle Keys, followed by inshore habitats of the Upper Keys (10.6°C). These two areas also endured the highest number of hours <16°C (140 and 110 hrs respectively), and had temperatures <12°C for 48 and 15 hrs respectively (Table 3). These temperature patterns correspond spatially with the mortality patterns documented for corals (i.e., highest in the Lower Keys and all inshore habitats within regions; Table 1, 2; Figure 3, S1). The examination of long-term temperature records for the FRT provided a historical context for the 2010 cold event [32]. The nearshore SEAKEYS station at Long Key had monthly means in January, February, and March 2010 that were statistically colder than in any prior year of sampling (1993–2010). The offshore SEAKEYS sites at Fowey Rocks and Molasses Reef also reached statistically significant cold extremes (vs. winter records for 1988–2010 and 1992–2010 respectively) during January through March.

**Table 3:** Sea temperature patterns recorded from representative reef sites during January 2010.

Subregion	Habitat	Min Temp (°C)	n hrs <16°C	n hrs <14°C	n hrs <12°C
Martin County	Inshore	13.4	54	26	0
Palm Beach County	Inshore	19.5	0	0	0
Broward County	Inshore	21.6	0	0	0
Biscayne	Inshore	12.8	85	11	0
	Mid-Channel	16.1	0	0	0
	Offshore	20.6	0	0	0
Upper Keys	Inshore	10.6	110	44	15
	Mid-Channel	15.1	2	0	0
	Offshore	15.9	1	0	0
Middle Keys	Inshore	9.5	140	78	48
	Mid-Channel	11.7	89	49	2
	Offshore	17.6	0	0	0
Lower Keys	Inshore	12.3	80	59	0
	Mid-Channel	14.5	55	0	0
	Offshore	18.7	0	0	0

Both the Degree-Cooling Days (DCD) and the ecoforecast stimulus/response index (S/RI) using site-based SST reproduced the spatial pattern of observed coral mortality (Figure 3, S2). The mean mortality exhibited by corals from the 76 sites surveyed in January-February 2010 was significantly related to number of DCD experienced at each site calculated based on satellite imagery (linear regression, r^2^ = 0.54, p<0.01). Mortality was also significantly related to the S/RI at each site (linear regression, r^2^ = 0.53, p<0.01).

## Discussion

The rapid response of public, scientific, and management stakeholders to dramatic natural disturbances in recent years has resulted in detailed characterizations of large-scale events that enhance our understanding of both causal factors and impacts, and the biological response (i.e., resistance and resilience) of ecosystems. Such an example was the characterization to the 2005 coral bleaching event that caused significant coral mortality world-wide. Immediately after this event, the impacts of elevated seawater temperatures on Caribbean coral reefs were assessed by >70 scientists and managers from 22 countries [38]. Due to the pressing need to learn from episodic disturbance events and to develop effective management and mitigation practices to preserve declining coral populations, a monitoring program now exists in Florida (the Florida Reef Resilience Program, FRRP). The FRRP brings together academic institutions, state and federal management trustees, and NGOs in a coordinated effort aimed at: (1) providing a multi-year baseline of coral reef condition against which the impacts of acute disturbances like elevated temperature events and hurricanes can be fully ascertained; and (2) maintaining the vested partnerships that can rapidly document the impacts of unusual disturbance events like the 2005 bleaching or the January 2010 cold-water events described herein. Whereas the 2005 summer bleaching event provided the impetus for this coordinated program, the winter of 2010 provided the opportunity to fully assess an unusual and highly detrimental event to coral reef communities.

The cold-water anomaly experienced by Florida reefs resulted in unprecedented, large-scale mortality of corals. Mortality was most catastrophic in shallow nearshore environments where temperatures were well below previously reported cold-water thresholds for extended periods of time during January 2010. While this is not the first report of cold-water coral mortality in Florida [20], [22]–[24], [39], this is the first time that coral mortality was recorded across such large spatial (Biscayne-Lower Keys) and taxonomic (84% of coral species experienced significant increases in tissue mortality compared to previous years) scales. The rapid, coordinated effort of numerous collaborators provided a unique opportunity to fully characterize this disturbance and compare its effects to those of warm-water anomalies on the same reef habitats over the previous five years. Coral bleaching caused by warm temperatures was documented in Florida in 2005, 2007, and 2009, but the mortality values recorded for most coral species after the 2010 cold-water event were 1–2 orders of magnitude higher than any observed in prior summers. Though the impacts of the cold event were only quantified here for stony corals, observations conducted by the collaborators of this study indicate that widespread mortality of other benthic organisms such as sponges and soft corals also occurred.

Unlike bleaching episodes where coral recovery can take place after temperatures return to normal [40], the cold-related impacts were extremely rapid and surveys conducted just weeks following the temperature minima already revealed severe coral tissue mortality. These observations are consistent with another study that showed rapid patterns of coral mortality in the Great Barrier Reef within six weeks of unusually cold winter conditions [11].

Research on the impacts of temperature anomalies on corals and coral reefs has concentrated almost exclusively on the effects of elevated temperatures. Warm anomalies have been documented as causal factors in the breakdown of the symbiotic relationship between coral hosts and zooxanthellae [9], [41] as well as in the increased prevalence of coral diseases [42], [43]. The physiological response of corals and their symbionts to temperature extremes may be similar for warm and cold-temperature anomalies [35], but this study shows that the impacts of the different types of temperature anomalies are species-specific and that taxa that showed high resistance to warm-water anomalies were especially affected by the cold water in the FRT.

The species affected significantly by the low temperatures did not represent a homogeneous group with respect to reproductive modes or colony morphology. Taxa with broadcasting (i.e., release gametes into the water columns) reproductive modes, mounding colony morphology, and large colony sizes (e.g., *Montastraea*) were equally affected as taxa like *Porites* that have brooding (i.e., internal fertilization) reproduction, branching or mounding morphology, and smaller colony sizes. Levels of tissue mortality were generally positively related to colony size and the mean size of colonies that experienced no tissue mortality was significantly smaller, for most species, than the mean size of colonies that did experience partial and total mortality. These findings are in agreement with previous field studies that showed that small colonies fare better during high-temperature anomalies, and that small colonies experience reduced bleaching and mortality compared to larger colonies of the same species in the same habitats [44], [45] This size-based pattern was predicted and explained theoretically by Nakamura and van Woesik [46] based on mass transfer and diffusion rates of gases and metabolites that are faster small organisms compared to large organisms. In situations when high mass-transfer rates are critical for the removal of toxins, such as periods of thermal stress [47], smaller colonies are thus predicted to fare better than larger ones.

The only coral taxa that did survive the cold event with limited mortality were those that are also found in other habitats that often experience low temperatures on a regular basis, suggesting species-specific adaptive resistance mechanisms. Examples of resistant taxa are *Solenastrea* and *Siderastrea,* dominant components of the shallow bays of South Florida where winter temperatures are commonly low [48] and are also found as far north as North Carolina where bottom temperatures of 10.6°C were documented [49], and *Oculina*, commonly found in deeper, colder shelf habitats [50]. Moreover, the only region that experienced low temperatures (minimum temperature = 13.4°C) but no significant coral mortality was the northermost region, Martin County, were coral abundance is already depressed due to seasonal temperature extremes and coral communities were dominated by species like *Oculina* and *Siderastrea* that have shown the highest resistance to cold temperatures in this study. Further research is clearly needed to evaluate the physiological traits that allow these species to survive in extreme temperature environments.

Inshore (i.e., closest to shore) patch-reef habitats in the FRT have been identified by previous research as among the healthiest reef habitats of the FRT, exhibiting significantly higher coral cover and coral growth rates compared to reef habitats located further offshore [51]. Additionally, these inshore habitats have a high abundance of the largest colonies of the reef-building genus *Montastraea* [52]. In fact, the presence of these large, “old-growth” colonies that often reach meters in diameter can be considered as evidence for the resistance and resilience of these habitats to natural and anthropogenic stressors (e.g., hurricanes, diseases, algal competition, vessel groundings) that have caused significant declines of offshore reefs in the same regions [53]–[55]. This pattern appears to be initially counter to the paradigm that good water quality is directly tied to reef condition as these habitats are known to experience higher nutrient and sedimentation conditions and more extreme temperatures compared to offshore reef habitats in the FRT [56]. While the reasons for this pattern are not clear, higher abundance of food availability (i.e., expanded nutritional niche) [57] and acclimation to fluctuating conditions may have played a role in the prior survivorship of these nearshore coral communities [51]. Unfortunately, the most severe mortality from the January 2010 cold-water event was concentrated on these last remaining oases where temperatures were the most extreme (reaching <10°C) and lasted longer than in deeper habitats further offshore. Moreover, the genus *Montastraea* proved to be the most susceptible taxon to the low temperatures (mean recent tissue mortality = 36.1% for all four species of this genus combined) and many of the largest and oldest colonies experienced 100% tissue mortality. The sexual recruitment of this genus has been extremely limited in the past decade [51], [58] and, thus, the loss of these important reef-building species represents a major setback to the ecology and future recovery of the FRT.

A potential explanation for the prior resilience and resistance of corals on inshore habitats is that, by being located in habitats that commonly experience wider fluctuations in environmental parameters compared to the more buffered and stable deeper habitats further removed from human influences, these corals may be acclimated or even adapted to environmental extremes. Prior exposure to wide fluctuations in temperature has been shown to confer some measure of resistance to corals exposed to rapid temperature increases [59], [60]. However, it is clear that the temperatures recorded during January 2010 exceeded low-temperature thresholds and overwhelmed any acclimatization advantages that most corals may have had in these nearshore environments.

Finally, the observed coral mortality patterns were strongly correlated with metrics of DCD and the S/RI developed during this study, thus they should both be considered by reef managers as novel remote sensing tools that can predict coral reef health during atypically cold weather events. These new tools could complement similar satellite-based metrics such as degree-heating weeks, which have been used extensively in the past to predict and describe coral bleaching and disease episodes during the warm-water anomalies [17], [38], [61], [62]. These remote sensing products are of significant value to assess large-scale response patterns synoptically, and they are especially valuable when paired by *in situ* ground-truthing activities to help identify areas, habitats, and populations at risk and in need of targeted conservation actions.

Coral populations around the globe have experienced drastic declines in abundance, extent, and condition over the recent past, and Florida reefs have mirrored these declining trends [18]. In the 2010 winter, the impacts of the cold water event negatively changed the structure and composition of many reefs of the FRT and reset the resilience trajectory of nearshore habitats that had been refugia to large-scale deterioration. This single event was one of the most destructive on record. The immediate ecological impacts of this disturbance were rapidly quantified, but the long-term impacts on services such as the valuable associated fisheries as well as recovery patterns will undoubtedly take years to decades to fully ascertain. In a time when coral reefs around the world are faced with increasing pressure from local stressors, the prospect of the potential synergistic impacts of factors commonly associated with Global Climate Change (GCC) is certainly troublesome for the long-term survivorship of these keystone resources. In the past, the concerns about GCC impacts on coral reefs have concentrated almost exclusively on increased-temperature scenarios (with ocean acidification becoming an increasingly important concern in the more recent past) and the impacts of warm-water coral bleaching [9]. However, as this study shows, coral reefs found at the limits of their low thermal thresholds, such as those in Florida, can also be significantly impacted by cold-water anomalies. While GCC scenarios predict a general warming trend and fewer cold days in the future [63], the frequency of extreme cold events may persist even under warming scenarios [64]. Thus, it is important that these types of events are also considered as significant threats to coral reefs, especially those found in marginal temperature environments.

While the mitigation of climatic anomalies may be beyond the scope of immediate management actions, there is a heightened recognition that for ecosystems to be able to survive large-scale disturbances such as those imposed by acute climatic events and, more importantly, global climate change, there is a pressing need to work at the local level to improve the resilience of threatened ecosystems. The reduction of land-based sources of pollution, the better management of renewable resources such as fisheries, and the implementation of management tools like Marine Protected Areas and active reef restoration, are all actions aimed at improving not only the present condition of coral reef communities, but also their resistance and resilience to future large-scale disturbances like temperature anomalies [65], [66]. In Florida, the protected status of the coral reefs in the area of influence of the cold-water anomaly (reefs in the Biscayne subregion are within a National Park and reefs in the Florida Keys are within a Marine Sanctuary) did little to mitigate the severe impacts of the cold, but the recovery of the affected habitats through colony re-growth and sexual recruitment will be likely influenced by the added levels of protection provided by these management jurisdictions.

## Materials and Methods

### Coral Surveys

A total of 76 reef sites (n = 3636 coral colonies) were surveyed between Jan 20–Feb 9, 2010 from the Lower Florida Keys to Martin County (Figure 1). Site selection followed a stratified-random survey design with subregion (Lower Keys, Middle Keys, Upper Keys, Biscayne, Broward, Palm Beach, Martin) and reef zones (inshore, mid-channel, offshore patch reef, and forereef) as main strata ([67]; Figure 1). At each site, divers surveyed the coral community along two 10-m^2^ belt transects following the FRRP survey protocol ([56]; http://frrp.org). Each coral colony ≥4 cm in diameter was identified to species, measured, and recent tissue mortality patterns evaluated as the percent of the surface area showing signs of mortality (Figure 2). Recent tissue mortality is defined as any non-living parts of the coral in which the corallite structures are still intact and identifiable to species [68]. Mortality patterns (mean % recent mortality) documented in 2010 were compared to data collected in similar fashion in the summers (Aug–Oct) of 2005–2009. A new set of random sites was selected each survey period.

### Temperature Patterns

*In situ* water temperature patterns were recorded using Onset HOBO Pro v2 underwater temperature loggers. In addition, long-term sea temperature records were obtained from the South Florida SEAKEYS/C-MAN network of *in situ* coral reef monitoring stations that have recorded hourly sea temperatures for approximately 20 years [69].

Satellite sea surface temperature (SST) data of the Advanced Very High Resolution Radiometer (AVHRR) and MODerate-resolution Imaging Spectroradiometer (MODIS) were obtained from ground stations and data archives managed by the Institute for Marine Remote Sensing (IMaRS) at the University of South Florida's College of Marine Science and from NASA archives. All data were processed to remove pixels obscured by clouds using a hybrid filtering algorithm [70]. Daily (05:00 to 05:00 GMT) composite images were created by calculating the mean of all SST measurements within each pixel for every day in January 2010. To elucidate spatial patterns in the reef habitats surveyed, daily composites were spatially oversampled to 200×200 m resolution using bilinear interpolation. Linear regression of daily composite satellite-derived SST data for January 2010 vs. coincident, *in situ* sea temperature data showed close agreement between these two data sources (p<0.05 for 9 out of 10 site-by-site comparisons).

For each coral site surveyed in January-February 2010 within the Biscayne and Florida Keys regions (where the coral mortality was concentrated), several SST-based metrics were calculated to relate temperature patterns to the observed coral mortality. These metrics included Degree-Cooling Days (DCD; e.g., [17], [71], [72]) and a numeric indicator for fuzzy-logic ecological forecasting known as the Stimulus/Response Index (S/RI; [17], [61]. DCD based on SST were calculated both for individual sites (pixels) and using the areal mean for each sub-region/zone. For each contiguous daily SST (pixel or areal mean) below 16°C, the DCD was increased cumulatively by (16°C - SST); for days above 16°C, DCD was reset to zero. The S/RI based on SST was constructed by assigning an S/RI value of 1.0 to each day between 14 and 16°C, 2.0 to each day between 14 and 12°C, and 2.5 to each day at or below 12°C. The greatest value (i.e., maximum DCD, cumulative S/RI) observed during January 2010 was used for comparison with coral mortality data.

## Supporting Information

Figure S1 **Mean percent tissue mortality of stony corals from the regions and habitats affected by the 2010 cold-water event.** OPR = Offshore Patch Reefs. Bold numbers represent mean depth (m)/mean distance to shore (km) for the sites surveyed within each habitat.
(TIF)

Figure S2 **Average satellite-derived cold-water metrics for the coral reef sites surveyed in January-February 2010.** (A) Degree-cooling days (DCD), (B) ecoforecast Stimulus/Response Index. OPR = Offshore Patch Reefs, values for these metrics were 0 for the forereef habitats in all four subregions.
(TIF)

Table S1 **Mean size of coral colonies that experienced no mortality and either total or partial mortality.**
(DOC)
