# Supplementary figures and images for: Severe 2010 Cold-Water Event Caused Unprecedented Mortality to Corals of the Florida Reef Tract and Reversed Previous Survivorship Patterns
Source: PLoS One. 2011 Aug 10;6(8):e23047. doi: 10.1371/journal.pone.0023047 (PMC3154280; doi:10.1371/journal.pone.0023047)

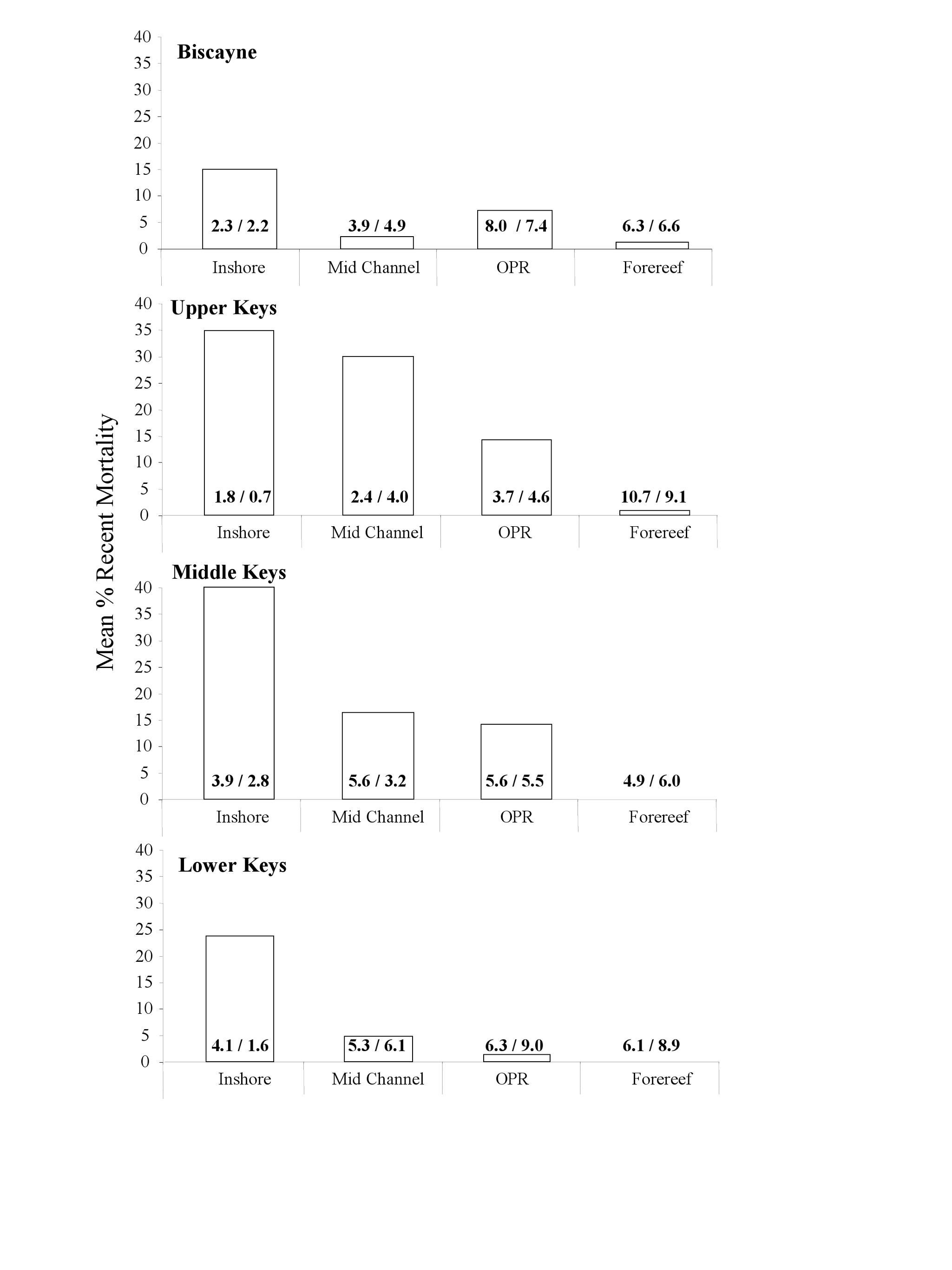

Supplement: Figure S1 — Mean percent tissue mortality of stony corals from the regions and habitats affected by the 2010 cold-water event. OPR = Offshore Patch Reefs. Bold numbers represent mean depth (m)/mean distance to shore (km) for the sites surveyed within each habitat. (TIF) [file pone.0023047.s001.tif]

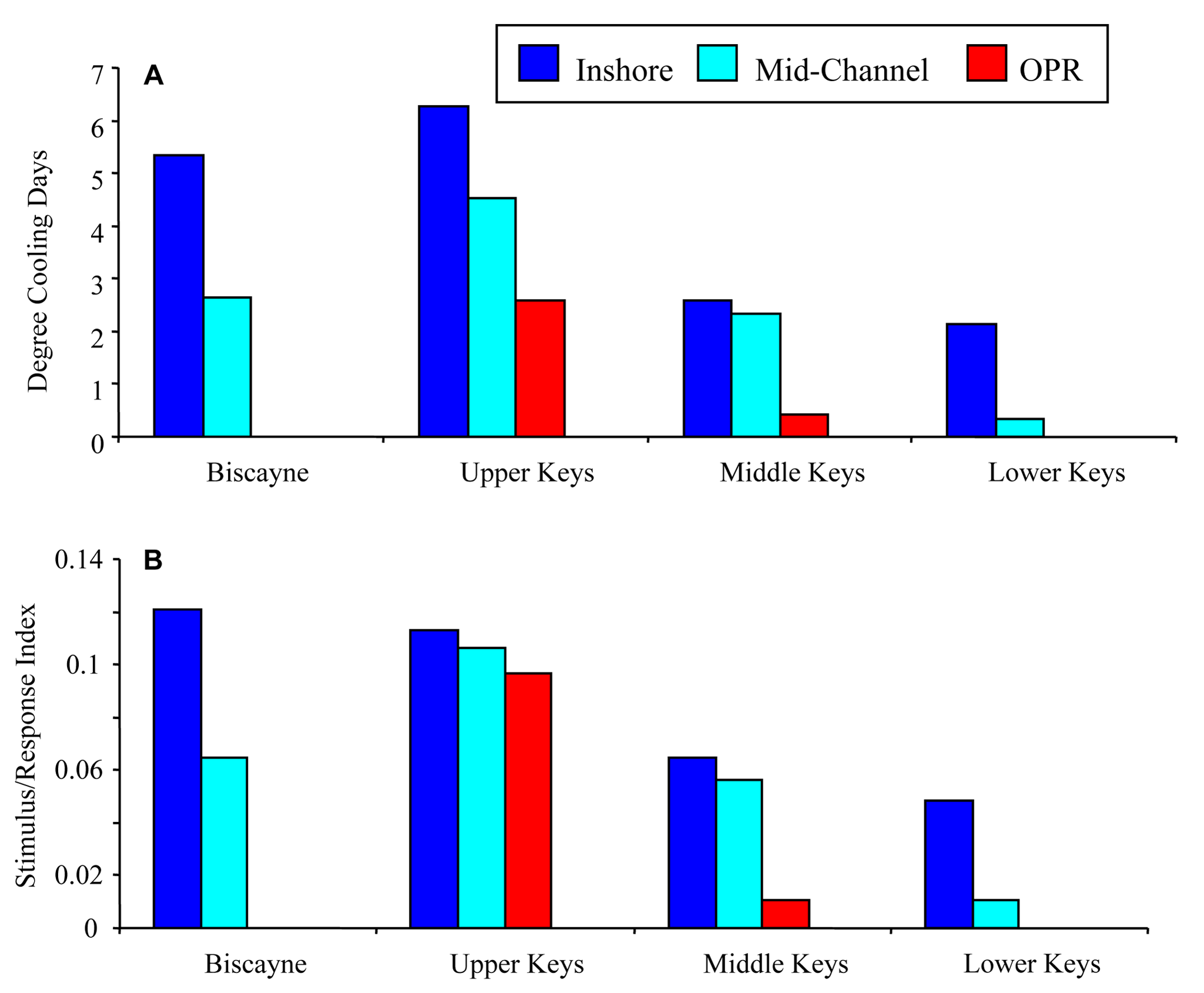

Supplement: Figure S2 — Average satellite-derived cold-water metrics for the coral reef sites surveyed in January-February 2010. (A) Degree-cooling days (DCD), (B) ecoforecast Stimulus/Response Index. OPR = Offshore Patch Reefs, values for these metrics were 0 for the forereef habitats in all four subregions. (TIF) [file pone.0023047.s002.tif]
