# Supplementary material for: Severe 2010 Cold-Water Event Caused Unprecedented Mortality to Corals of the Florida Reef Tract and Reversed Previous Survivorship Patterns
Source: PLoS One. 2011 Aug 10;6(8):e23047. doi: 10.1371/journal.pone.0023047 (PMC3154280; doi:10.1371/journal.pone.0023047)
Supplement: Table S1 — Mean size of coral colonies that experienced no mortality and either total or partial mortality. (DOC) [file pone.0023047.s003.doc]

**Table S1.** Mean size of coral colonies that experienced no mortality and either total or partial mortality.

|  | Mean Colony Size (cm, ± SD) | | p value |
| --- | --- | --- | --- |
| Species | No Mortality | Partial or Total Mortality |
| *Agaricia agaricites* | 10.2 (4.9) | 12.6 (5.9) | 0.05 |
| *Colpophyllia natans* | 50.4 (46.8) | 46.7 (34.7) | 0.7 |
| *Diploria clivosa* | 17.9 (18.7) | 33.8 (26.4) | 0.02 |
| *Dichocoenia stokesi* | 14.2 (9.9) | 21.4 (11.0) | 0.02 |
| *Diploria strigosa* | 28.6 (19.9) | 50.1 (40.3) | 0.01 |
| *Montastraea annularis* | 65.8 (72.1) | 64.9 (52.9) | 0.44 |
| *Montastraea cavernosa* | 28.8 (28.0) | 54.1 (43.6) | <0.001 |
| *Montastraea faveolata* | 67.0 (57.6) | 90.0 (71.1) | 0.08 |
| *Porites astreoides* | 11.6 (6.8) | 15.0 (10.8) | <0.001 |
| *Porites furcata* | 13.5 (7.3) | 18.9 (12.8) | 0.08 |
| *Porites porites* | 13.9 (12.1) | 16.6 (15.1) | 0.23 |
| *Solenastrea bournoni* | 18.8 (13.4) | 24.8 (9.5) | 0.24 |
| *Stephanocoenia intersepta* | 13.4 (8.6) | 18.0 (11.0) | 0.01 |
| *Siderastrea radians* | 11.3 (8.3) | 19.6 (15.3) | 0.03 |
| *Siderastrea siderea* | 19.5 (17) | 28.0 (28.2) | 0.01 |

able S1. 15 most abundant specieshigher levels of tissue mortality compared to larger coloniesy tissue mortality (Table S1). o

p values correspond to the results of a t-test comparing average colony sizes between mortality categories for each species. Only the 15 most abundant species are included here.
